# Incidence of elevated intraocular pressure after intravitreal injection in Japanese patients with age-related macular degeneration

**DOI:** 10.1038/s41598-021-91832-w

**Published:** 2021-06-10

**Authors:** Maiko Maruyama-Inoue, Tatsuya Inoue, Shaheeda Mohamed, Yoko Kitajima, Shoko Ikeda, Arisa Ito, Kazuaki Kadonosono

**Affiliations:** 1 Department of Ophthalmology, Yokohama City University Medical Center, 4-57 Urafune-cho, Minami-ku, Yokohama, Kanagawa 232-0024 Japan; 2 Department of Ophthalmology and Visual Sciences, Hong Kong Eye Hospital, The Chinese University of Hong Kong, Hong Kong Special Administrative Region, Hong Kong, China; 3 Department of Ophthalmology, Kanto Rosai Hospital, Kawasaki, Japan

**Keywords:** Diseases, Medical research

## Abstract

The purpose of this study was to report the incidence of elevated intraocular pressure (IOP) after intravitreal injection (IVI) of anti-vascular endothelial growth factor (VEGF) in Japanese patients with age-related macular degeneration (AMD). A retrospective study of chart review of patients who underwent ≥ 10 intravitreal anti-VEGF injections between April 2009 and December 2019 was conducted. Elevated IOP was defined as IOP ≥ 25 mmHg at one visit. Cases with elevated IOP resulting from IVI were identified. Furthermore, the association between elevated IOP and some parameters, as the risk factors that influence elevated IOP, was investigated. A total of 402 eyes of 370 patients were included in this study. Twenty-eight eyes of 26 patients (7.0%) were identified as cases with elevated IOP after IVI. The mean time of elevation after baseline was 50.6 ± 26.5 months. History of glaucoma (*p* = 0.021; odds ratio, 5.85), treatment modality (*p* = 0.019; odds ratio, 6.32), and total number of injections (*p* = 0.003; odds ratio, 1.03) were significantly associated with elevated IOP. A late complication of elevated IOP is associated with IVI in patients with AMD. Particularly, history of glaucoma and treat and extend regimen with frequent injections were found to be risk factors of elevated IOP.

## Introduction

Age-related macular degeneration (AMD) is the leading cause of severe irreversible vision loss in older adults in the United States and other developed countries^1, 2^. In Japan, the prevalence rate of neovascular AMD is approximately 0.67%, and patients with neovascular AMD has a male predominance, which is contradictory to population-based studies in the Caucasian population^3–5^. The current standard in the diagnosis of neovascular AMD relies on multimodal imaging, including fluorescein angiography, indocyanine green angiography, and optical coherence tomography (OCT)^6, 7^.

Neovascular AMD is mainly treated with intravitreal injection of anti-vascular endothelial growth factor (VEGF), which has shown efficacy in improving visual acuity outcomes^8, 9^. Regarding the treatment modality, various regimens are used. Treat and extend (TAE) regimen, which is placed as a proactive treatment regimen, is based on the intervals of recurrent disease. Then, intervals of treatment are determined to minimize the need for checking patients. In contrast, as needed regimen (pro re nata; PRN) can reduce the frequency of repeated intravitreal injections although it needs frequent monitoring.

It is known that intraocular pressure (IOP) transiently increases from a volume effect after intravitreal injection; however, this generally decreases and is well tolerated by most patients^10^. On the contrary, some studies showed that elevated IOP is associated with repeated intravitreal injections of anti-VEGF agents^11, 12^. However, the incidence of elevated IOP after intravitreal anti-VEGF injections has not yet been reported in Japan. This study aimed to investigate the incidence of elevated IOP following intravitreal injection in Japanese patients with neovascular AMD.

## Results

A total of 402 eyes of 370 patients were enrolled in the study. Demographic and clinical characteristics of patients with neovascular AMD are presented in Table 1. Of 370 patients, there were 250 (67.6%) men and 120 (32.4%) women. The mean patient age was 80.1 ± 7.4 years (median, 80 years; range, 58–99 years). The mean baseline IOP was 15.1 ± 2.6 mmHg. During the study period, the mean number of anti-VEGF injections administered was 27.7 ± 18.6. The mean follow-up period was 73.3 ± 27.8 months. Among the 402 eyes, 300 eyes were phakic, and 102 eyes were pseudophakic.

**Table 1:** Clinical characteristics of the patients with neovascular AMD.

Number of patients	370
Number of eyes	402
Age, mean ± SD, year	80.1 ± 7.4
Sex (male/female) (%)	250 (67.6)/120 (32.4)
Baseline mean IOP in treated eyes (mmHg)	15.1 ± 2.6
Baseline mean IOP in untreated fellow eyes (mmHg)	15.2 ± 2.6
Mean number of anti-VEGF injections	27.7 ± 18.6
Mean follow-up period (month)	73.3 ± 27.8
Lens status (phakic/pseudophakic) (%)	300 (74.6)/102 (25.4)

*AMD* age-related macular degeneration, *SD* standard deviation, *IOP* intraocular pressure, *VEGF* vascular endothelial growth factor.

Twenty-eight eyes of 26 patients (7.0%) showed elevated IOP after intravitreal injection. The comparison of clinical characteristics between patients who showed elevated IOP and patients who did not show elevated IOP are presented in Table 2. There were no significant differences in sex, age, and history of glaucoma between the two groups (all, *p* > 0.05). However, patients who showed elevated IOP tended to have higher baseline IOP, TAE treatment, phakic eyes, large number of treatment, and longer follow-up period (all, *p* < 0.05). Moreover, 25 eyes were well controlled by using topical medications. In contrast, elevated IOP decreased spontaneously without medication in the remaining three eyes. The mean period from baseline to the time of higher IOP was 50.6 ± 26.5 months.

**Table 2:** Comparison of the clinical features between patients with elevated IOP and normal IOP.

	Elevated IOP (n = 28)	Normal IOP (n = 374)	*p* value*
Number of patients	26	345	
Sex (male/female)	17/9	234/111	0.829
Age, mean ± SD, year (range)	80.0 ± 6.6	80.6 ± 7.4	0.670
History of glaucoma (+/−)	3/25	12/362	0.078
Mean baseline IOP	16.1 ± 2.4	15.1 ± 2.6	0.047
Treatment modality (TAE/PRN)	26/2	216/158	< 0.001
Lens status (phakic/pseudophakic)	25/2	275/99	0.036
Mean number of injection	45.1 ± 25.3	26.4 ± 17.4	< 0.001
Follow-up period (month)	83.6 ± 23.5	72.5 ± 28.0	0.024

*IOP* intraocular pressure, *SD* standard deviation, *TAE* treat and extend, *PRN* pro re nata.

*p value calculated using Welch’s t-test and the fisher’s exact test.

In 338 fellow eyes, 45 eyes were treated with < 10 intravitreal injections of anti-VEGF agents. Therefore, the remaining 293 untreated fellow eyes were compared with treated 402 eyes. In 293 untreated fellow eyes of 293 patients, two eyes (0.7%) showed elevated IOP during the follow-up period. Both eyes were fellow eyes that showed elevated IOP. Moreover, the period in which the IOP increased was almost the same as that in the treated eyes. The treated eyes showed significantly higher rate of elevated IOP compared with the untreated fellow eyes (*p* < 0.001).

Table 3 shows the results of the stepwise logistic analyses. History of glaucoma (*p* = 0.021; odds ratio, 5.85; 95% confidence interval, 1.299–26.340), treatment modality (*p* = 0.019; odds ratio, 6.32; 95% confidence interval, 1.352–29.510), and total number of injections (*p* = 0.003; odds ratio, 1.03; 95% confidence interval, 1.009–1.044) were significantly associated with elevated IOP. However, no significant correlations were seen between other explanation variables examined in this study (age, sex, lens status, and follow-up period) and elevated IOP (all *p* > 0.05).

**Table 3:** Logistic analyses which investigate factors influencing elevated IOP.

Variable	Multivariate analysis		
	Odds ratio	95% CI	*p* value
History of glaucoma	5.85	1.299–26.340	0.021
Treatment modality (TAE or PRN)	6.32	1.352–29.510	0.019
Lens status	0.30	0.082–1.066	0.067
Total number of treatment	1.03	1.009–1.044	0.003

Excluded variables: age, sex, follow-up period.

*IOP* intraocular pressure, *CI* confidential interval, *TAE* treat and extend, *PRN* pro re nata.

## Discussion

Our retrospective investigation demonstrated that an increase in IOP after intravitreal injection of anti-VEGF agents occurs at an estimated incidence rate of 7.0%, which was significantly higher than that of untreated fellow eyes. Although elevated IOP following anti-VEGF injection is recognized to be a rare complication, we should consider that history of glaucoma, TAE regimen, and frequent injections were the risk factors of elevated IOP. This is the first study to describe the changes in IOP after anti-VEGF in Japanese patients with neovascular AMD with mean follow-up period of > 6 years.

Previous studies showed that repeated intravitreal anti-VEGF injections have been associated with ocular hypertension (OHTN), which was consistent with our study. Although the definition of sustained OHTN was varied, some studies reported that sustained OHTN developed in 3.4–11.0% of eyes with AMD^13–16^. In contrast, some studies found no association between sustained elevation of IOP and factors such as total number of injections, interval between injections, and preexisting glaucoma^11, 17, 18^. These results were controversial; however, the lack of an effect of intravitreal injections on long-term IOP in some studies may be due to the small number of injections and short follow-up periods. The strengths of our study are the large number of patients with longer follow-up. In fact, the mean period of elevated IOP was 50.6 ± 26.5 months from the baseline, which indicates that an increase in IOP could occur as a late complication of repeated anti-VEGF injections even several years after the first injection.

Although the mechanism of elevated IOP after anti-VEGF injections is still unclear, various studies support that intravitreal injections may influence the pressure-dependent outflow system of the trabecular meshwork. One proposed mechanism is related to microparticle obstruction of the trabecular meshwork^19^. Some studies have described that silicone microdroplets and protein aggregates from a medication package or delivery equipment can obstruct aqueous outflow^20–22^. Furthermore, a previous study demonstrated that subclinical inflammation after intravitreal injection can cause scar formation and fibroblast proliferation in the trabecular meshwork, which result in chronic obstruction with impaired aqueous outflow^23^. Another proposed mechanism is that repeated, long-term anti-VEGF injection may lead to a deficiency in intraocular nitric oxide, which affects outflow and leads to a sustained elevation of IOP^24^. Moreover, several studies demonstrated that repeated trauma and/or immediate IOP spikes associated with the injection procedure may cause damage to the trabecular meshwork^11, 13, 20, 25, 26^. In this study, most patients who showed elevated IOP possibly controlled their IOP by medication use. It is uncertain that elevated IOP decreased spontaneously without medication use in three eyes. However, elevated IOP in some patients might be reversible, and there may be a transient elevation in these three eyes.

In this study, history of glaucoma, TAE treatment, and larger number of injections were significantly associated with elevated IOP by logistic analyses. Although having phakic eyes and longer follow-up also lead to elevated IOP as shown in the univariate analysis, these factors were not selected in the multivariate analysis. Previous studies demonstrated that the total number of injections was a risk factor for sustained OHTN, which was consistent with this study^12, 16^. Wen et al. found a statistically significant decrease in outflow facility in patients with > 20 intravitreal injections compared with those with < 10 injections^27^, which supports the result that the total number of injections is a risk factor for elevated IOP. However, in this study, the odds ratio of total number of treatment was relatively low that the influence to the elevated IOP might be less than treatment modality and history of glaucoma. Moreover, Mathalone et al. described that shorter intervals between injections, especially within 8 weeks, was a risk factor for IOP elevation^15^. This result is similar to the finding of our study that TAE regimen was related to elevated IOP. There is no clear consensus about the mechanism of IOP elevation due to TAE regimen; however, regular injections may induce repeated subclinical inflammation or damage of the trabecular meshwork, which result in impaired outflow capacity. In contrast, in patients receiving PRN regimen, anti-VEGF is cleared before the next injection with minimum damage to the outflow capacity; therefore, patients receiving PRN regimen might have a low risk of IOP elevation. On the contrary, history of glaucoma was also reported as a risk factor for elevated IOP, which was consistent with our study^11^. Wen et al. suggested that patients with both increased number of anti-VEGF injections and underlying impaired outflow capacity are at increased risk of further reduction in outflow facility^27^. We speculate that patients with history of glaucoma might have underlying impaired outflow capacity; therefore, these patients might show elevated IOP.

In our study, 0.7% of untreated fellow eyes showed elevated IOP during the follow-up period. Both eyes were fellow eyes that showed elevated IOP after anti-VEGF treatment. We speculate that patients who showed elevated IOP after intravitreal injections tended to have the originally underlying pathological outflow capacity even in the untreated fellow eyes. Anti-VEGF is known to be measured systemically following intravitreal injection^28^. Therefore, treated eyes could have effects on the untreated fellow eyes. Although systemic exposure of anti-VEGF should be extremely low and the degree of the effect to the fellow eyes would be little, it might cause elevated IOP even in the untreated fellow eyes after repeated anti-VEGF treatment.

There are several limitation in this study. A major limitation is its retrospective design. However, its strength lay on the analyzed cases with large number of injection and long follow-up period; the consecutive patient series may have minimized this study weakness. Moreover, in this study, we did not consider the association between elevated IOP and anti-VEGF agents used because anti-VEGF agents were switched in most patients on the way of the treatment and difficult to evaluate. Two anti-VEGF agents used in this study had various molecular size and might influence the results. Furthermore, whether nerve fiber layer (NFL) damage may have occurred is also important in patients with elevated IOP. We need to perform NFL measurements or visual fields and evaluate the damage resulting from intravitreal injections in the future.

In conclusion, there is a late complication of elevated IOP associated with intravitreal injection in patients with neovascular AMD. Particularly, a history of glaucoma, TAE regimen, and frequent injections were found to be risk factors of elevated IOP. Although intrinsic weakness of outflow capacity might be related to elevated IOP, visual acuity in patients with neovascular AMD should be maintained by repeated injections. Therefore, it might be difficult to decrease the number of treatment or change their treatment modality. Fortunately, this study shows that elevated IOP was well controlled by medication or decreased spontaneously. We should consider that patients who were treated with anti-VEGF agents, especially with history of glaucoma and TAE treatment regimen with frequent number of injections, have a high risk of elevated IOP and monitor them carefully.

## Methods

We retrospectively studied consecutive Japanese patients with neovascular AMD to investigate the incidence of elevated IOP following intravitreal anti-VEGF injection. We evaluated patients who underwent intravitreal injections of ranibizumab (Lucentis; Novartis Pharma AG, Basel, Switzerland, and Genentech Inc., South San Francisco, CA, USA), and aflibercept (Eylea; Bayer HealthCare, Berlin, Germany). All patients were treated between April 2009 and December 2019 at the Yokohama City University Medical Center in Japan. The study was conducted according to the principles of the Declaration of Helsinki, and informed consent was obtained from all the patients. The study was conducted with the approval of the ethics committee of Yokohama City University Medical Center.

The inclusion criteria were the diagnosis of neovascular AMD based on clinical and angiographic findings, which was treated with ≥ 10 intravitreal injections of anti-VEGF agents, and availability for follow-up in December 2019. Patients who had previously underwent vitrectomy, intravitreal injection of any other anti-VEGF agent, and intravitreal or subtenon steroid administration were excluded from the study.

All patients received three consecutive monthly intravitreal injections of 0.5 mg/0.05 mL of ranibizumab or 2 mg/0.05 mL of aflibercept through the pars plana via a 30-G needle as an induction treatment. For patients treated with PRN, all patients were instructed to receive monthly follow-up examination after the loading phase. The intravitreal injection was repeated in cases of persistence or recurrence of subretinal fluid, intraretinal edema, or fluctuation of pigment epithelial detachment on the SD-OCT images or clinically detectable macular hemorrhages, as judged by the retina specialist (M.M). Patients with TAE regimen received loading doses of three initial monthly injections followed by continued treatment of 0.5 mg/0.05 mL of ranibizumab, or 2 mg/0.05 mL of aflibercept. Treatment intervals were increased by 1 week per visit if SD-OCT findings or macular hemorrhage, as mentioned above, were not observed and decided based on the recurrence period. The maximum tolerated interval is then maintained. Intravitreal injections were administered through the pars plana 3.5–4 mm posterior to the limbus using a 30-gauge needle.

IOP was measured on every visit using the Goldmann applanation tonometer. Elevated IOP was defined as IOP ≥ 25 mmHg at one visit.

Cases of elevated IOP were collected by a medical record, and the data of these patients were summarized. The proportion of treated eyes with elevated IOP was compared with that of untreated fellow eyes. Furthermore, the association between elevated IOP and parameters, including age, sex, lens status, history of glaucoma, treatment modality (TAE or PRN regimen), total number of injections, and follow-up period, as the risk factors that influence elevated IOP, was investigated. Moreover, the interval from baseline to presentation of elevated IOP and the treatments were subsequently reviewed.

The incidence rate of elevated IOP was calculated by dividing the number of cases with elevated IOP by the total number of injected cases. Fisher’s exact test was used to compare the proportion of treated eyes and untreated eyes with elevated IOP. Factors influencing elevated IOP were analyzed using stepwise logistic analyses. *p* values < 0.05 were considered to indicate statistical significance.
